# Prognostic Value of Baseline Serum Pro-Inflammatory Cytokines in Severe Multisystem Inflammatory Syndrome in Children

**DOI:** 10.3390/jcm13237177

**Published:** 2024-11-26

**Authors:** Anita Bartha-Tatár, György Sinkovits, János Schnur, Veronika Maráczi, Máté Dávid, Borbála Zsigmond, Éva Rimanóczy, Balázs Szalay, Edina Biró, Gabriella Bekő, Petra Varga, Tamás Szabó, Miklós Fagyas, Zsolt Fejes, János Kappelmayer, Béla Nagy Jr.

**Affiliations:** 1 Department of Laboratory Medicine, Faculty of Medicine, University of Debrecen, 4032 Debrecen, Hungary; tatar.anita@med.unideb.hu (A.B.-T.); fejes.zsolt@med.unideb.hu (Z.F.); kappelmayer@med.unideb.hu (J.K.); 2 Doctoral School of Kálmán Laki, Faculty of Medicine, University of Debrecen, 4032 Debrecen, Hungary; 3 Department of Internal Medicine and Hematology, Semmelweis University, 1088 Budapest, Hungary; 4 Heim Pál National Pediatric Institute, 1089 Budapest, Hungary; schnurjanos@gmail.com (J.S.); marver78@gmail.com (V.M.); krounk.david@gmail.com (M.D.); borbala.zsigmond@gmail.com (B.Z.); erimanoczy@heimpalkorhaz.hu (É.R.); 5 National Institute of Hematology and Infectious Disease, 1097 Budapest, Hungary; szalay.balazs@dpckorhaz.hu (B.S.); biro.edina@dpckorhaz.hu (E.B.); beko.gabriella@dpckorhaz.hu (G.B.); 6 Institute of Pediatrics, Faculty of Medicine, University of Debrecen, 4032 Debrecen, Hungary; peatra81@gmail.com (P.V.); szabotamas@med.unideb.hu (T.S.); 7 Department of Cardiology, Division of Clinical Physiology, Faculty of Medicine, University of Debrecen, 4032 Debrecen, Hungary; fagyasmiklos@med.unideb.hu

**Keywords:** SARS-CoV-2, MIS-C, cytokine, inflammation, biomarker, IL-18, TNF-α, disease severity

## Abstract

**Background:** Severe clinical manifestations of multisystem inflammatory syndrome in children (MIS-C) are associated with the dysregulation of immune response following SARS-CoV-2 infection. Therefore, we analyzed the levels of 10 selected cytokines at admission to estimate disease severity and to predict the length of hospitalization. In remission samples, these mediators were followed after intravenous immunoglobulin (IVIG) treatment before discharge. **Methods:** Thirty-five MIS-C patients at the age of 8.4 ± 4.1 years and 11 clinical controls were included. Acute MIS-C patients were divided into two severity subgroups based on their clinical score determined by the WHO criteria. Serum concentrations of IFN-γ, IL-1α, IL-1RA, IL-8, IL-10, IL-17A, IL-18, IP-10, MCP-1, and TNF-α were measured by MILLIPLEX^®^ Human Cytokine/Chemokine panel, while ACE2 activity was determined by a fluorescent kinetic assay. These results were correlated with routinely determined laboratory parameters and clinical characteristics. **Results:** MIS-C patients demonstrated significantly elevated baseline levels of most of these cytokines compared to controls. Even higher concentrations of IL-18, TNF-α and ferritin with reduced lymphocyte count were found in severe subjects with elevated clinical scores of 4–5 compared to moderate cases with a clinical score of 1–3. Furthermore, the development of cardiovascular dysfunction and prolonged hospitalization (≥8 days) were related to augmented ACE2 and IL-6 levels. IL-18, IL-1RA, IL-10 and TNF-α were diminished in response to IVIG treatment in remission samples. Finally, pre-treatment IL-18 (≥516.8 pg/mL) and TNF-α (≥74.2 pg/mL) effectively differentiated disease severity in MIS-C with AUC values of 0.770 and 0.750, respectively. **Conclusions:** IL-18 and TNF-α have a prognostic value in disease severity at admission and are capable of monitoring the efficacy of IVIG treatment in MIS-C.

## 1. Introduction

Multisystem inflammatory syndrome in children (MIS-C) is a rare, potentially life-threatening complication of an infection with the severe acute respiratory syndrome coronavirus 2 (SARS-CoV-2). This disease typically occurs 2–6 weeks after exposure to the virus and is characterized by hyperinflammatory conditions and cardiogenic shock that overlap with Kawasaki disease, or toxic shock syndrome [1]. Patients demonstrate a wide range of signs and symptoms, including persistent fever and mucocutaneous lesions with gastrointestinal and cardiogenic complications [2]. Hence, there is a clinical need to promptly recognize the excess of inflammation and subsequent multiorgan failure, as well as to manage hospital interventions. Approximately 60% of these patients are admitted to an intensive care unit (ICU), and mortality can be as high as 2% [3]. Understanding the pathomechanism of the exaggerated immune response in MIS-C is still under investigation. Based on recent data, the dysregulation of the immunological events consists of unregulated lymphocyte activation, immune complex-mediated cell activation, complement activation by autoantibodies, and related endothelial cell damage, which may propagate the development of critically ill conditions [4].

Laboratory tests are highly implicated in the routine clinical examination of MIS-C, as a variety of laboratory parameters support the diagnosis and evaluation of disease severity. The increased inflammatory state during the acute phase of MIS-C is characterized by highly elevated serum levels of acute phase proteins, e.g., C-reactive protein (CRP), interleukin-6 (IL-6), or procalcitonin (PCT). In addition, myocardial dysfunction and damage can be monitored via cardiac troponin T or I (cTnT/I) and N-terminal pro-B-type natriuretic peptide (NT-proBNP), while typical hematologic alterations like reduced absolute lymphocyte and platelet (PLT) counts are often observed in these children. Evidence of coagulopathy—i.e., prolonged prothrombin time (PT) and/or activated partial thromboplastin time (APTT) with elevated D-dimer levels—and evidence of a recent SARS-CoV-2 infection by RT-PCR, serology, or antigen test are also among the criteria for the MIS-C diagnosis according to the World Health Organization (WHO) [5].

Some recent clinical studies reported that levels of routinely not measured pro-inflammatory cytokines, such as IL-2, IL-10, IL-23, and interferon gamma-induced protein 10 (IP-10), were significantly elevated in severe SARS-CoV-2 infection in adults compared to controls; these were associated with disease severity [6,7,8]. However, only limited data are available on the baseline expression and prognostic value of such cytokines in MIS-C [9,10]. Furthermore, the immunological features of MIS-C subjects overlapped with those in pediatric patients with severe COVID-19 [11]. Therefore, the main aim of the present study was to examine a set of cytokines at admission in serum samples of patients with acute severe MIS-C. These biomarkers were chosen based on their substantial alteration and favorable clinical application in adults with acute severe COVID-19 [6,12]. We hypothesized that pretreatment levels of selected pro-inflammatory cytokines could be useful in evaluating the clinical severity of MIS-C and monitoring the efficacy of intravenous immunoglobulin (IVIG) treatment.

## 2. Materials and Methods

### 2.1. MIS-C Patients and Clinical Controls

Thirty-five children at the age of 8.4 ± 4.1 years (21 males and 14 females) admitted to two major clinical centers (University of Debrecen, Debrecen, Hungary and Heim Pál National Pediatric Institute, Budapest, Hungary) from December 2020 to October 2022 who had been diagnosed with MIS-C were included into this retrospective study. These subjects were enrolled during the 5 different waves of the COVID-19 pandemic as follows: 1st and 2nd wave (March 2020–January 2021), with the Wuhan variant: 21 patients; 3rd wave (February–July 2021) with the alpha-variant: 8 patients; 4th wave (July–December 2021) with the delta variant: 1 patient; and 5th wave (December 2021–October 2022) with the omicron variant: 5 patients. They met the following Centers for Disease Control and Prevention (CDC) 2020 MIS-C definition criteria: (1) patient’s age < 21 years, (2) clinically severe illness requiring hospitalization, (3) no alternative diagnosis, (4) fever (≥1 day), (5) laboratory evidence of inflammation, (6) evidence of SARS-CoV-2 infection or exposure, and (7) multisystem (≥2) organ involvement (cardiovascular, renal, respiratory, hematologic, gastrointestinal, mucocutaneous, neurologic) [13]. Recent SARS-CoV-2 infection was confirmed by the presence of specific autoantibodies in serum, and no patient had received anti-SARS-CoV-2 vaccination prior to their disease. These patients demonstrated several types of symptoms at different ratios and were classified into the following categories: WHO A (rash/conjunctivitis/mucocutaneous inflammation): 91%; WHO B (hypotension/shock): 69%; WHO C (myocardial dysfunction/pericarditis/valvulitis/coronary abnormalities): 77%; WHO D (coagulopathy: INR > 1.2/APTT > 35.0 sec): 74%, however, no acute thrombotic complications occurred; and WHO E (gastrointestinal problems): 91%. In addition, 37% of the children suffered from pulmonary disorders as well. All patients required hospitalization: 20 patients (57%) were transmitted to the ICU at admission, with a median hospitalization length of 5.2 days (min-max: 1–100 days), while 15 were monitored in the Pediatric Ward. The WHO criteria were used to determine the clinical score for the severity of disease according to the organ systems involved. The presence of the following disorders increased the overall clinical score by 1 point: (1) rash or bilateral non-purulent conjunctivitis or muco-cutaneous inflammation signs (oral, hands or feet), (2) hypotension or shock, (3) features of myocardial dysfunction, pericarditis, valvulitis, or coronary abnormalities (including ECHO findings or elevated cTnT/I or NT-proBNP), (4) evidence of coagulopathy (by PT, APTT, elevated D-dimer), and (5) acute gastrointestinal problems (diarrhoea, vomiting, or abdominal pain) [14]. The maximum clinical score was 5 points for the most severe clinical manifestations. Recruited patients were divided into 2 subgroups based on their clinical score at admission: moderate cases with clinical scores of 1–3, and patients with clinical scores of 4–5, showing severe clinical manifestations (Table 1). MIS-C-specific treatment, the degree of pulmonary involvement based on chest X-ray/CT, administration of invasive ventilation, the length of hospital stay, and the clinical outcome were recorded by the clinicians for all patients. All patients received IVIG therapy, corticosteroids, anticoagulant (LMWH), and anti-platelet therapy (aspirin) during hospitalization, while 70% of children treated at the ICU were administered with cardiovascular support as well. In terms of disease outcome, each subject recovered, and thus no death occurred among these study participants.

As clinical controls, 11 age- and gender-matched children (8.4 ± 5.2 years of age, 7 males, 4 females) with mild non-COVID-19 viral infections were recruited based on their having negative Rapid COVID-19 Antigen Test or RT-PCR test results and some mild complaints (i.e., headache, earache, upper airway symptoms, dorsalgia, etc.). These subjects were treated at the Pediatric Outpatient Clinic of the University of Debrecen for an average of 3 days.

### 2.2. Laboratory Analyses

All study participants had baseline peripheral venous blood samples drawn at admission prior to any MIS-C related treatment (before immunomodulatory therapy). Follow-up/remission samples were available before discharge from the hospital in the case of 26 patients after a median of 39.5 (IQR: 13–167) days of admission. In controls, an acute blood sample obtained at admission was involved for comparison. Routinely available laboratory tests were performed at the Departments of Laboratory Medicine, University of Debrecen and Semmelweis University, while serum concentrations of selected cytokines were retrospectively measured at the Research Laboratory, Department of Internal Medicine and Hematology, Semmelweis University, Budapest, Hungary. Baseline and follow-up sera were stored at −70 °C and the analysis of serum cytokines (IFN-γ, IL-1α, IL-1RA, IL-8, IL-10, IL-17A, IL-18, IP-10, MCP-1, and TNF-α) were measured retrospectively via a MILLIPLEX^®^ Human Cytokine/Chemokine panel (Sigma-Aldrich, St. Louis, MO, USA) using a MAGPIX^®^ instrument (Merck, Darmstadt, Germany). In parallel, the analysis of serum ACE2 activity was performed by a specific quenched fluorescent substrate (obtained from http://peptide2.com accessed on 16 September 2022) as reported earlier [15].

Routinely available laboratory serum tests, i.e., CRP, PCT, IL-6, ferritin, cTnT, and NT-proBNP, were determined using electro-chemiluminescent immunoassays on a Cobas^®^ e411 analyzer (Roche Diagnostics, Mannheim, Germany), while enzyme activities (i.e., AST, ALT, LD) were analyzed via kinetic colorimetric assays on a Cobas^®^ 8000 instrument (Roche Diagnostics). D-dimer was measured using a Coagulation Analyzer ACLTOP^®^ 550 CTS (Werfen, Bedford, MA, USA) using an immunoturbidimetric assay. In parallel, hematology parameters, i.e., white blood cell (WBC) and PLT count, absolute lymphocyte count, and hemoglobin concentration were determined with an Advia^®^ 2120 Hematology System analyzer (Bayer Diagnostics, Tarrytown, NJ, USA). Total SARS-CoV-2 S-specific antibody titers were quantified by a Cobas^®^ Anti-SARS-CoV-2 S serology test (Roche Diagnostics). The test consisted of an electro-chemiluminescence indirect assay and included two recombinant RBD antigens, which bound serum antibodies in a double-antigen sandwich setup. Total anti-SARS-CoV-2 S-specific antibody results measured in U/mL were converted to the WHO international unit based on a user circular provided by the manufacturer (U/mL = 0.972 × BAU/mL). Seropositivity was evaluated based on the manufacturer’s cut-off values of 0.8 BAU/mL.

### 2.3. Statistical Analyses

The Kolmogorov–Smirnov test was used for evaluation of the normality of data. Results are expressed as median with the interquartile range (IQR) or mean ± standard deviation (SD), as appropriate. To compare the data of the two groups, we applied the Mann–Whitney U test and the chi-squared test. Correlations between cytokine levels and other laboratory parameters were determined using Spearman’s test. Serum cytokine values in baseline and follow-up samples were compared with each other using the Wilcoxon matched pairs signed rank test. The area under the receiver operating characteristic curve (ROC-AUC) value was determined for each baseline cytokine to indicate the severity of the MIS-C. The maximum of the Youden index was determined to identify the cut-off values. We used GraphPad Prism software (version 8.01, GraphPad Software Inc., La Jolla, CA, USA) for the statistical calculations. Statistical significance was considered if the *p* value was < 0.05.

## 3. Results

### 3.1. Baseline Characteristics of MIS-C Patients

Baseline serum samples obtained in a total of 35 acute MIS-C patients were analyzed to measure a set of different cytokines at hospital admission to estimate disease severity. For this purpose, these patients were divided into two subgroups according to their MIS-C severity score. Regarding age and sex, there was no difference between MIS-C subgroups with respective clinical scores of 4–5 and 1–3. Based on the routine laboratory tests at admission, general inflammatory markers, such as CRP (*p* = 0.030) and ferritin (*p* = 0.010), were significantly higher, whereas PLT (*p* = 0.030) and absolute lymphocyte count (*p* < 0.001) were lower in severe patients (clinical score: 4–5) compared to moderate cases (clinical score: 1–3). Of note, IL-6 showed a ‘borderline’ significant difference between the two severity subcohorts (*p* = 0.058). In terms of coagulopathy, D-dimer values were significantly higher in MIS-C patients with severity scores of 4–5 compared to the less severe patient group (*p* = 0.001), indicating a trend toward hypercoagulation; however, no acute thrombotic events occurred in any of these patients (Table 1).

### 3.2. Alteration in Pro-Inflammatory Cytokines in MIS-C with Different Disease Severity

Next, baseline serum cytokine levels and ACE2 activity were determined for each MIS-C and control patient to evaluate which biomarkers were abnormal in this patient group. Serum IFN-γ, IL-1RA, IL-8, IL-10, IL-17A, IL-18, IP-10, MCP-1 and TNF-α levels were significantly higher in patients with MIS-C compared to clinical controls (*p* < 0.001 or *p* < 0.0001, respectively) (Table 2). When we further analyzed these cytokines to differentiate disease severity already at hospital admission, severe MIS-C individuals had even higher baseline IL-18 (*p* = 0.016) and TNF-α (*p* = 0.031) levels than the moderate subcohort. In addition, IL-1RA concentrations differed with a statistically marginal significance (*p* = 0.0535) between the two severity subgroups. Interestingly, serum ACE2 activity was not significantly elevated in the entire MIS-C group vs. controls and showed no difference between severe and moderate cases either (*p* = 0.066) (Table 2). Overall, increased production of these cytokines was independent of anti-SARS-CoV-2 humoral immune response as no significant difference was found in serology results between the two severity subcohorts (Table 1).

### 3.3. Association Between Inflammation Dependent Biomarkers and Disease Progression in MIS-C

**Figure 1:**
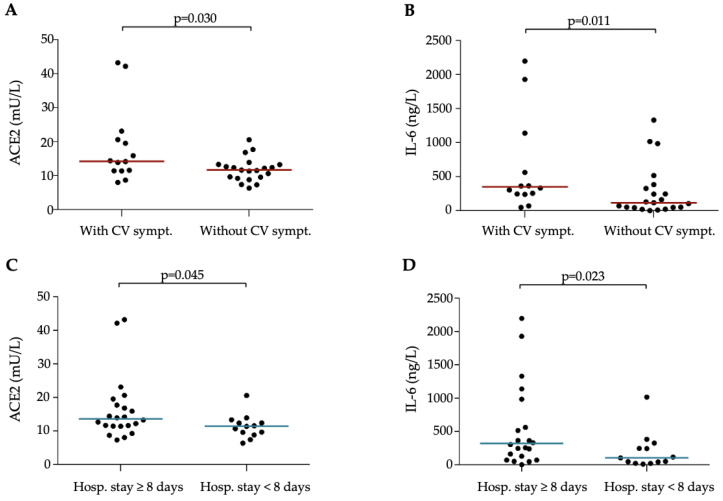
Comparison of baseline serum ACE2 activity and IL-6 concentration in MIS-C patients based on disease severity. Dots represent single results, while bars indicate median value. To compare the data of the two groups, the Mann–Whitney U test was applied. Abbreviations: CV sympt: cardiovascular symptoms, Hosp. stay: hospital stay, IL-6: interleukin-6, ACE2: angiotensin converting enzyme 2.

Abnormal levels of variable laboratory parameters were further examined in relation to the presence of cardiovascular complications and length of hospital stay in this MIS-C group (Figure 1A–D). Patients who developed severe cardiovascular symptoms had significantly higher baseline ACE2 activity (*p* = 0.030) and IL-6 concentration (*p* = 0.011) than those without cardiovascular symptoms. In the case of subjects whose hospital stay lasted more than 8 days, significantly higher ACE2 activity (*p* = 0.045) and serum IL-6 levels (*p* = 0.023), as well as WBC count (*p* = 0.013) at admission, were determined compared to those discharged earlier. Furthermore, we sought to observe if any of the newly measured cytokines could differentiate MIS-C patients with distinct clinical complications, such as those who required CV support, had WHO-B or WHO-C symptoms, or underwent longer hospitalization. Although there was a tendency toward higher baseline levels in the more severe subjects, surprisingly, none of these biomarkers (e.g., IL-10, IP-10, IFN-γ etc.) showed a significant difference during these comparisons, probably due to the relatively low number of cases (Supplementary Figure S1).

**Figure 2:**
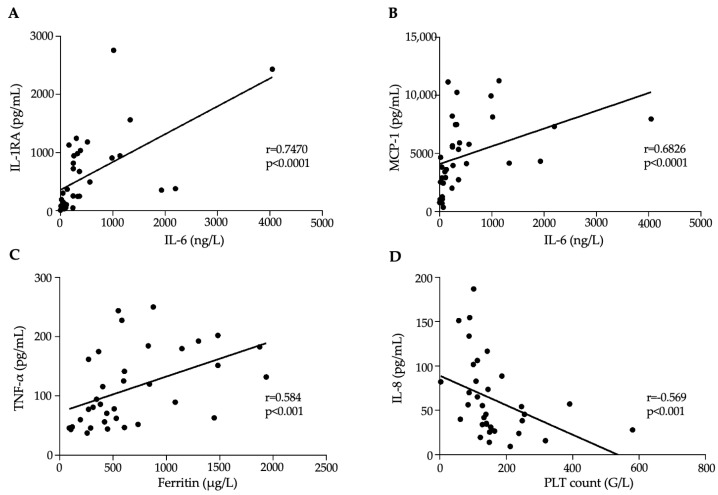
Correlation between baseline routine laboratory parameters and cytokine levels. IL-6, ferritin, and PLT count were significantly correlated with IL-1RA (**A**), MCP-1 (**B**), TNF-α (**C**), and IL-8 (**D**), respectively. Dots represent single results. Correlations between cytokine levels and other laboratory parameters were determined using Spearman’s test.

Next, we investigated significant correlations with an “r” value of > 0.5 between routinely determined laboratory parameters and serum cytokine levels (Supplementary Table S1). Our findings underlined that IL-6 was significantly correlated with ACE2, IL-1RA, IL-10, MCP-1, and TNF-α, while ferritin was associated with ACE2, IP-10, and TNF-α. Furthermore, PCT was related to IL-1RA, IL-18, and TNF-α, while PLT count showed a negative association with IL-1RA, IL-8, IL-18, IL-10, and TNF-α. Some of the most significant correlations are depicted in Figure 2A–D.

**Figure 3:**
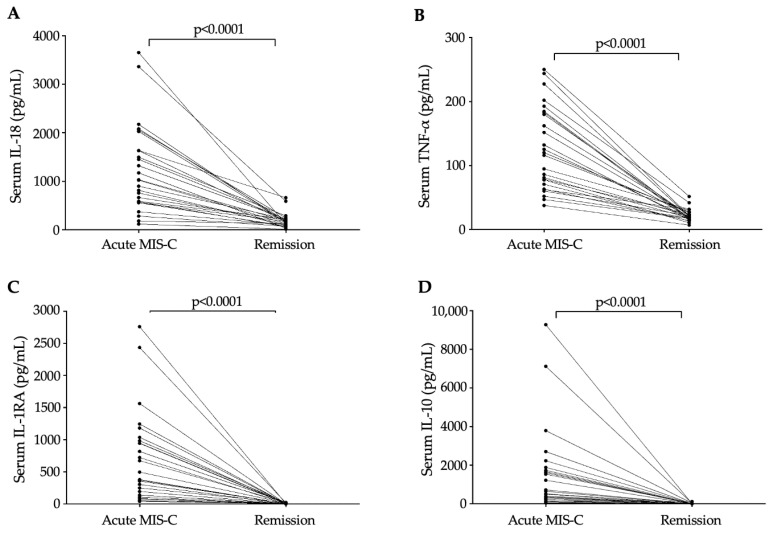
Changes in different cytokine levels in all MIS-C patients in response to IVIG treatment. Dots represent single results, while lines connect cytokine values measured in baseline and remission samples. The degree of alterations in pro-inflammatory cytokine values were analyzed using the Wilcoxon matched-pairs signed rank test.

Regarding clinical outcome, all recruited MIS-C patients recovered, and no deaths occurred in the follow-up period. Remission samples were available in the case of 26 MIS-C individuals; thus, we could monitor the change in cytokine levels in parallel with treatment before discharge. There was a significant reduction in the level of all serum cytokines under IVIG treatment compared to baseline values. However, the largest alteration was observed in IL-18, TNF-α, IL-1RA, and IL-10 among these subjects before and after IVIG treatment (*p* < 0.0001) (Figure 3A–D).

### 3.4. Diagnostic Efficacy of Serum Cytokines to Assess Disease Severity in MIS-C

**Figure 4:**
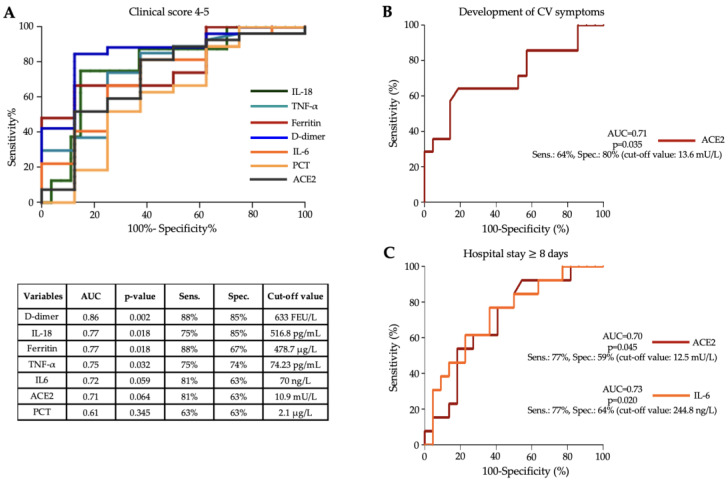
ROC-curve analysis for the comparison of selected baseline cytokine levels and renowned prognostic markers for the assessment of disease severity (**A**), cardiovascular involvement (**B**), and in the prediction of length of hospital stay (**C**) in MIS-C disease. AUC values with *p*-values as well as sensitivity and specificity values were determined using ROC curve analysis for each parameter.

To evaluate the effectiveness of baseline level of distinct serum cytokines mentioned above in MIS-C, we statistically analyzed their diagnostic characteristics to estimate disease severity and cardiovascular involvement or to predict the length of hospital stay (Figure 4). For this purpose, ROC-AUC curve analyses were performed for each parameter. According to these analyses, IL-18, TNF-α, ACE2, and IL-6 showed clinically the most substantial power for one of the required purposes. The best discriminative threshold of IL-18 and TNF-α levels at admission to estimate disease severity, based on the Youden index, was 516.8 pg/mL with a sensitivity of 75% and a specificity of 85% at an AUC value of 0.77 (*p* = 0.018) and 74.23 pg/mL with a sensitivity of 75% and a specificity of 74% at an AUC value of 0.75 (*p* = 0.032), respectively (Figure 4A). In addition, a cut-off value for indicating the severity of MIS-C disease was also established for routine laboratory parameters: D-dimer (at cut-off value of 633 FEU/L, AUC: 0.86, *p* = 0.002) and ferritin (at cut-off value: 478,7 μg/L, AUC: 0.77, *p* = 0.018) were still the most effective, while IL-6 with an AUC value of 0.72, PCT (AUC: 0.61) and ACE2 activity (AUC: 0.71) were inferior to IL-18 and TNF-α (Figure 4A). To assess cardiovascular involvement, the ideal cut-off value of baseline ACE2 activity was 13.6 mU/L with a sensitivity of 64%, a specificity of 80%, and with an AUC value of 0.71 (*p* = 0.035) (Figure 4B). When a longer (≥8 days) length of hospital stay was tested to be predicted, the optimal threshold for baseline ACE2 activity was 12.5 mU/L, showing a sensitivity of 77% and a specificity of 59% with an AUC value of 0.70 (*p* = 0.045), while the best discriminative threshold of serum IL-6 level was 244.8 ng/L, showing a sensitivity of 77% and a specificity of 64% with an AUC value of 0.73 (*p* = 0.020) (Figure 4C). Based on these results, among the newly investigated cytokines, baseline IL-18 and TNF-α were effective in assessing the progression of MIS-C disease similar to D-dimer and ferritin.

## 4. Discussion

MIS-C is a rare, life-threating complication of SARS-CoV-2 infection that may rapidly disseminate into hyperinflammatory syndrome, severe myocarditis, or shock, thus requiring ICU interventions [3]. It typically occurs in children at the age of 8 to 12 years several weeks after exposure to an often-mild SARS-CoV-2 infection and clinical features include fever, rash, conjunctivitis, mucositis, hypotension, and cardiac complications [16]. Recent studies have described the pathomechanism of immunological dysregulation in MIS-C, including highly activated neutrophils, monocytes/macrophages, B-plasma blasts with T lymphocyte depletion and activation, and decreased numbers and functional profiles of antigen-presenting cells. In addition, autoantibodies are produced at large quantities against several antigens, such as endothelial targets, causing vascular abnormalities [17]. Furthermore, acute MIS-C has recently been characterized by activated classical, alternative, and terminal complement pathways leading to elevation of C1s-C1-inhibitor complex and C4a and C4d levels, increased Bb and C3a concentrations, and elevation of sC5b-9, which were independent of anti-SARS-CoV-2 humoral immune response [18]. Current treatment for MIS-C includes supportive care, vasoactive medication in combination with immunomodulatory treatment. Optimal treatment is still unknown; however, the most frequently used agent is IVIG with or without corticosteroids, resulting in the rapid resolution of inflammation [19,20].

Laboratory tests are highly involved in the clinical diagnosis of MIS-C, and several routinely available laboratory parameters support the evaluation of disease severity [6,7,8,21]. However, only limited data are available on the baseline expression and prognostic value of pro-inflammatory cytokines in MIS-C [9,10]. Hence, in this study, we investigated the serum levels of 10 different mediators involved in the development of hyperinflammatory response in MIS-C in a total of 35 unvaccinated patients treated in two clinical centers to identify potential new biomarkers of disease severity. These cytokines were selected based on their substantial alteration and favorable clinical application in adults with acute severe COVID-19 [6,12]. We hypothesized that pretreatment levels of these parameters could be useful in estimating the clinical severity of MIS-C and monitoring the effect of IVIG treatment at remission.

We recruited samples of the first series of MIS-C cases in Hungary [18,22] but they are being investigated from new perspectives. These patients demonstrated several types of symptoms at different ratios and were classified into the following categories: rash/conjunctivitis/mucocutaneous inflammation: 91%; hypotension/shock: 69%; myocardial dysfunction/pericarditis/valvulitis/coronary abnormalities: 77%; coagulopathy: 74%; and gastrointestinal problems: 91%. In addition, 37% of the children suffered from pulmonary disorders as well. These ratios were identical to previous studies from other countries [2,3,4,19,23]. Twenty patients (57%) were transmitted to the ICU at admission with a median hospitalization length of 5.2 days, while 15 were monitored in the Pediatric Ward. Based on their clinical score, children were divided into two subgroups: moderate cases with clinical scores of 1–3, and patients with clinical scores of 4–5, showing severe clinical manifestations (Table 1). Those subjects with higher clinical scores had a larger requirement for ICU treatment (74%) and showed even higher CRP, D-dimer, and ferritin, as well as lower absolute lymphocyte and platelet counts at admission than those with moderate clinical score values. Similarly, in former studies, ICU admission was more likely for patients with highly increased concentrations of CRP, troponin, ferritin, D-dimer, NT-proBNP, and IL-6, or reduced platelet or lymphocyte counts [3,24]. Interestingly, IL-6 showed a ‘borderline’ significant difference between our severity subcohorts. Although D-dimer values were significantly higher in MIS-C patients with severity scores of 4–5 compared to the less severe patient group, which was a sign of hypercoagulation, no acute thrombotic events occurred in any of these patients. In a meta-analysis, BNP but not troponin, was found to be an important cardiac marker distinguishing between severe and less severe MIS-C subjects [25].

Although in our study a marked difference was observed in its serum concentrations between the two patient subgroups, our results did not show a significant difference in this aspect, presumably due to the relatively small sample size. Previous reports also implied the importance of different routinely not measured cytokines in laboratory diagnostics of severe SARS-CoV-2 infection induced clinical conditions [11]. For instance, robust inflammatory response to MIS-C with elevated IL-1β, IFN-α, GM-CSF, and HMGB1 levels explained the severity and outcome of the clinical syndrome in both MIS-C and COVID-19 disease [26], while the serum concentrations of IL-18 were correlated with creatinine, liver enzymes, and troponin, which reflected disease severity in COVID-19 disease with macrophage activation syndrome [27]. Moreover, serum ACE2 activity has been found to be clinically relevant to predicting disease severity and mortality in hospitalized COVID-19 adult patients [15]. Hence, we determined baseline serum cytokine levels and ACE2 activity for each MIS-C patient in contrast to non-COVID-19 controls to evaluate which biomarkers were abnormal in this patient group. Serum IFN-γ, IL-1RA, IL-8, IL-10, IL-17A, IL-18, IP-10, MCP-1, and TNF-α levels were significantly higher in patients with MIS-C compared to clinical controls (Table 2). Overall, increased production of these cytokines was independent of anti-SARS-CoV-2 humoral immune response, as no significant difference was found in serology results between the two severity subcohorts (Table 1). Non-significant elevation of IL-1α in MIS-C was opposite to the results of other groups, where the authors described highly augmented values, especially in male children [28]. In terms of ACE2, according to our knowledge, no former data were available on MIS-C to compare with our current findings. Here, serum ACE2 activity was not significantly elevated in the entire MIS-C group vs. controls and showed no difference between severe and moderate cases. On the other hand, higher serum ACE2 activity and IL-6 levels in MIS-C were associated with severe cardiovascular involvement and longer hospital stay. Recently, serum ACE2 activity did not differ either between SARS-CoV-2-infected pregnancies and age- and gestational age-matched healthy controls to predict COVID-19 placentitis and threatening fetal loss [29]. Meanwhile, the predictive value of IL-6 on cardiac manifestations, among several other cytokines and chemokines, such as CCL2, CCL3, CCL11, and CXCL10 (IP-10), was reported in MIS-C [30]. Next, we further analyzed these cytokines to differentiate disease severity upon hospital admission, severe MIS-C individuals with high clinical scores (4–5) had even higher baseline IL-18 and TNF-α levels than those with moderately increased severity scores (1–3). In addition, IL-1RA concentrations differed with a statistically marginal significance between the two severity subgroups. We also performed a logistic regression analysis in which all routinely measured and newly analyzed parameters with demographic and clinical data were considered to globally study which biomarker was the most reliable marker to predict disease severity. Interestingly, only absolute lymphocyte count showed a significant odds ratio of 0.824 (*p* = 0.016) in terms of MIS-C severity. One potential reason can be that the age, clinical score, and laboratory parameters tightly moved together diminishing their individual prognostic values in MIS-C.

Subsequently, we used ROC-AUC curve analyses to test the predictive value of serum levels of various cytokines and routine laboratory parameters for the severity of MIS-C. The best discriminative thresholds for predicting disease severity were serum levels of IL-18 and TNF-α at hospital admission of 516.8 pg/mL and 74.23 pg/mL, with AUC values of 0.770 and 0.750, respectively. Satış et al. found a strong association with an increased risk of ICU admission for acute COVID-19 infection at serum IL-18 levels > 576 pg/mL [27]. No threshold for serum TNF-α levels in MIS-C was found in the literature; however, several studies claimed that TNF-α discriminated between patients with MIS-C and severe COVID-19 [31] or differentiated MIS-C children with cardiac manifestations from those without cardiac manifestations [30]. The importance of analyzed cytokines above is also highlighted by a special treatment approach applied in MIS-C. Cytokine-removing hemofilter representing a 100 h-extracorporeal blood purification therapy resulted in a marked reduction of levels of several cytokines, including TNF-α, IL-6, IL-8, and IL-10, which greatly improved the clinical conditions of an MIS-C patient with decreased serum ferritin, CRP, and PCT levels, leading to favorable clinical progress [32]. Others tested the usefulness of the sepsis marker soluble triggering receptor expressed on myeloid cell-1 (sTREM-1) as a potential biomarker in MIS-C. ROC curve analysis revealed a significant AUC of 0.870, and the sTREM-1 cut-off value of >5781 pg/mL yielded a sensitivity of 71.4% and a specificity of 91.3% for disease severity, and patients with sTREM-1 levels above this cut-off presented an elevated risk for MIS-C development of 22.85-fold [33].

One of the strengths of this study was that we also collected follow-up/remission samples that were available in 26 MIS-C patients after (median) 39.5 days of admission. There was a significant reduction in the level of all serum cytokines under IVIG treatment compared to baseline values. However, the largest alterations were observed in IL-18, TNF-α, IL-1RA, and IL-10 among these subjects before and after IVIG treatment. In agreement with our findings, significantly reduced cytokine concentrations have been reported after treatment in patients with acute COVID-19 receiving IVIG therapy [34]. For instance, increased levels of cytokines, such as IFN-γ, IL-2, TNFα, IL-5, IL-1α, IL-1β, IL-6, IL-10, and IL-12p70, as well as chemokines CCL2, CCL3, CCL11, and CXCL10 (IP-10), in MIS-C children with cardiac manifestations significantly diminished and normalized 9 months after treatment [30]. In addition, decreased complement activation was closely associated with rapid improvement of MIS-C after IVIG treatment [18]. Of note, the pronounced inflammatory response underscores its involvement in acute organ damage, while its 12-month persistence suggests potential implications for long-term metabolic disorders [35].

In conclusion, these data emphasize that the measurement of serum cytokines at admission may be useful for predicting the outcome of MIS-C. We found that baseline IL-18 and TNF-α have a prognostic value in disease severity and are capable of monitoring IVIG treatment efficacy in MIS-C.

## 5. Limitations

Our study has some limitations. This was a retrospective, non-randomized clinical study, and clinical data were also collected retrospectively. However, our study was designed to ensure that all study participants had evidence of prior SARS-CoV-2 infection, follow-up samples were collected from most of the involved patients, a matched clinical control group was applied, and careful analytical measurements were performed on a large set of cytokines. The number of MIS-C patients included in this study was low (n = 35), but due to the collection of 26 acute (before immunomodulatory therapy) and remission sample pairs with appropriate serum aliquots for cytokines, this is among the largest pediatric studies reported to date with similar measurements. The effect sizes between the healthy controls and the severe as well as moderate MIS-C subgroups were large enough to yield sufficient power and strongly significant differences, even with such low case numbers. The healthy control group was used to provide estimates of normal values of pro-inflammatory cytokines. However, we did not have a sufficiently large diseased clinical control group; therefore, we were not able to determine whether the described changes are specific to MIS-C. Cytokine expression profile in MIS-C needs to be interpreted with caution depending on the preanalytical conditions, such as the type of samples, e.g., serum or plasma, and fresh or defrosted whole blood samples, which may modulate the results. Overall, further studies are required to evaluate the diagnostic potential of pro-inflammatory cytokines in MIS-C.

## Figures and Tables

**Table 1:** Baseline demographical and routine laboratory characteristics of 35 MIS-C patients and 11 clinical controls.

Patient Parameters	Clinical Controls (n = 11)	Total MIS-C Patients (n = 35)	MIS-C Patients with Score 4–5 (n = 27)	MIS-C Patients with Score 1–3 (n = 8)	*p*-Value (Score 4–5 vs. Score 1–3)
**Sex** (M/F)	7/4	21/14	18/9	3/5	0.220
**Age** (years)	8.4 ± 5.2	8.4 ± 4.1	8.9 ± 3.6	6.5 ± 5	0.210
**WBC count** (G/L)	7.8 (7.1–8.6)	10.11 (7.07–13.6)	9.77 (6.8–12.7)	11.87 (8.9–22.7)	0.080
**Hemoglobin** (g/L)	137 (119–142) *	116 (107–123)	115 (105–123)	116 (112.5–145.8)	0.360
**PLT count** (G/L)	327 (282–374) ***	140 (102–187)	133 (101–153)	229 (133.5–372.8)	0.030
**Lymphocyte count** (G/L)	2.8 (2.2–3.4) ***	1 (0.6–1.6)	0.8 (0.4–1.4)	2.36 (1.5–4.5)	<0.001
**CRP** (mg/L)	0.6 (0.5–0.6) ***	152.4 (112.1–215.3)	164.8 (117.6–244.4)	99.69 (20.72–167)	0.030
**PCT** (µg/L)	n.m.	2.64 (0.8–7.6)	3.19 (0.9–7.6)	1.82 (0.3–7.6)	0.350
**IL-6** (ng/L)	n.m.	244.8 (50.9–515.2)	255.8 (102.3–561.2)	60.32 (26.4–290)	0.058
**Ferritin** (µg/L)	49.3 (20.7–51.1) ***	529.2 (312.7–876.6)	599.4 (344.8–1146)	392.5 (108–445.8)	0.010
**LDH** (U/L)	222 (217–266.5)	390 (220.8–566.3)	402.5 (262.8–609.3)	322.5 (116.8–466)	0.240
**AST** (U/L)	23 (22.5–26) *	33 (25–80)	35 (26–80)	28.5 (18–83.2)	0.430
**ALT** (U/L)	16 (7.2–18.2) *	28 (16–58)	32 (20–58)	13.17 (11.2–85)	0.140
**cTnT** (ng/L)	n.m.	24 (9.2–54.5)	28.15 (9.2–49.8)	10 (4.7–153.4)	0.760
**D-dimer** (FEU/L)	n.m.	1102 (18–2684)	1779 (856.5–2831)	4.6 (1.2–352)	0.001
**NT-proBNP** (ng/L)	n.m.	2087 (573.4–8309)	2454 (490–12460)	1440 (611.1–4285)	0.410
**Anti-Spike-SARS-Cov2 total Ig** (BAU/mL)	n.m.	500 (194.9–854.6)	500 (194.9–802.5)	611.2 (160.4–19110)	0.490

Data are expressed as mean ± SD or median with IQR. The WHO classification was used to determine the clinical score for disease severity based on the number of affected organs. Study participants were divided into two subgroups based on disease severity scores. For statistical analyses, the Mann–Whitney U test or Fisher’s exact test was used, as appropriate. Abbreviations: CRP: C-reactive protein, PCT: procalcitonin, IL-6: interleukin-6, AST: aspartate transaminase, ALT: alanine aminotransferase, LD: lactate dehydrogenase, WBC: white blood cell, PLT: platelet, cTnT: cardiac troponin T, NT-proBNP: N-Terminal Pro–B-Type Natriuretic Peptide, n.m. means ‘not measured’. * *p* < 0.05, *** *p* < 0.001 in comparison between all MIS-C patients vs. clinical controls.

**Table 2:** Ten different cytokines and serum ACE2 activity in 35 MIS-C patients and 11 control participants.

Variables	Clinical Controls (n = 11)	Total MIS-C Patients (n = 35)	MIS-C Patients with Score 4–5 (n = 27)	MIS-C Patients with Score 1–3 (n = 8)	*p*-Value (Score 4–5 vs. Score 1–3)
**IFNγ** (pg/mL)	5.1 (3.9–5.9)	47.49 (19.7–76.5) ***	40.38 (16.1–76.5)	48.68 (21.4–179.8)	0.961
**IL1-α** (pg/mL)	136 (122.1–170.1)	155.2 (132.1–202.5)	166.4 (136–202.5)	131.1 (103.6–196.6)	0.102
**IL1-RA** (pg/mL)	8.93 (4.9–12.1)	357.4 (107.3–947.3) ***	380.4 (139.8–985)	120 (36.9–744.2)	0.053
**IL-8** (pg/mL)	19.38 (13.1–22.5)	45.65 (28–82.9) **	45.65 (34.1–101.8)	41.18 (18.3–56.8)	0.138
**IL-10** (pg/mL)	42.7 (16.9–81.7)	519.3 (152.7–1668) ***	654 (282.9–1740)	265.3 (23–1434)	0.110
**IL-17A** (pg/mL)	3.85 (2.1–6.8)	10.49 (8–17.9) **	11.1 (8–17.9)	9.41 (6.2–17)	0.410
**IL-18** (pg/mL)	158.8 (121.3–401.6)	760 (469.9–1557) **	1025 (581.4–1637)	446 (307–672.3)	0.016
**IP-10** (pg/mL)	61.43 (35.39–101)	14713 (1666–47008) ***	15707 (6578–53,166)	1481 (1047–38,934)	0.100
**MCP-1** (pg/mL)	811.2 (641.3–924)	4174 (2542–7486) ***	4336 (2937–7317)	2721 (909.9–10,180)	0.558
**TNF-α** (pg/mL)	20.57 (15.4–27.3)	89.55 (56.3–174.8) ***	116 (63.15–182)	52.08 (44.84–123.8)	0.031
**ACE2 activity** (mU/L)	11.62 (9.53–16.25)	12.34 (9.6–15.9)	13.26 (11.41–16.83)	9.92 (7.72–12.53)	0.066

Data are expressed as median with IQR. For statistical analyses, the Mann–Whitney U test was used. Significant differences were found in different comparisons indicated with variable symbols as follows: *** *p* < 0.0001, ** *p* < 0.001 in case of comparison between all MIS-C patients and controls. Abbreviations: IFNγ: interferon-gamma, IL1-α: interleukin-1 alpha, IL-1RA: interleukin-1 receptor antagonist, IL-8: interleukin-8, IL-10: interleukin-10, IL-17A: interleukin-17A, IL-18: interleukin-18, IP-10: interferon gamma-induced protein 10, MCP-1: monocyte chemoattractant protein 1, TNF-α: tumor necrosis factor alpha, ACE2: angiotensin converting enzyme 2.

## Data Availability

The raw data supporting the conclusions of this article will be made available by the authors on request.

## Supplementary Materials

The following supporting information can be downloaded at: https://www.mdpi.com/article/10.3390/jcm13237177/s1: Figure S1. Comparison of baseline serum IL-10, TNF-α, ACE2, IP-10, and IFN-γ concentration in MIS-C patients based on the need for CV support (A,B), WHO-B symptoms (C), WHO-C symptoms (D) and the length of hospitalization (E,F). Table S1. Correlation analysis between baseline routine laboratory parameters and pro-inflammatory cytokine levels using Spearman’s test.
