# Supplementary material for: Prognostic Value of Baseline Serum Pro-Inflammatory Cytokines in Severe Multisystem Inflammatory Syndrome in Children
Source: J Clin Med. 2024 Nov 26;13(23):7177. doi: 10.3390/jcm13237177 (PMC11642126; doi:10.3390/jcm13237177)
Supplement: Supplementary file 1 [file jcm-13-07177-s001.zip › jcm-3266742-supplementary.pdf]

Supplementary materials

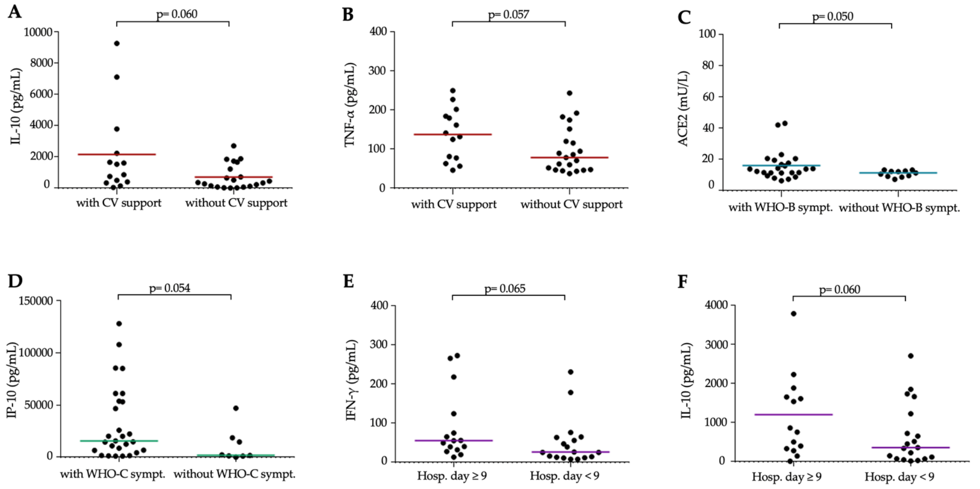

Suppl. Figure 1. Comparison of baseline serum IL-10, TNF-α, ACE2, IP-10, and IFN-γ concentration in MIS-C patients based on the need for CV support (A-B), WHO-B symptoms (C), WHO-C symptoms (D) and the length of hospitalization (E-F). Dots represent single results, while bars indicate median value. To compare the data of two groups, Mann-Whitney U test was applied. Abbreviations: CV support: cardiovascular support, WHO-B/C sympt: WHO-B/C symptoms, Hosp. stay: hospital stay, IL-6: interleukin-6, ACE2: angiotensin converting enzyme 2

Suppl. Table 1. Correlation analysis between baseline routine laboratory parameters and pro-inflammatory cytokine levels using Spearman's test.

Variables		r	p
IL-6	ACE2	0.518	0.001
	IFN-γ	0.471	0.004
	IL-1RA	0.7470	<0.0001
	IL-8	0.3571	0.035
	IL-10	0.5319	0.001
	IL-17A	0.3374	0.048
	IP-10	0.3597	0.034
	MCP-1	0.6826	<0.0001
	TNF-α	0.6591	<0.0001
Ferritin	ACE2	0.546	<0.001
	IL-1RA	0.405	0.016
	IL-8	0.461	0.005
	IL-10	0.359	0.034
	IP-10	0.576	<0.001
	TNF-α	0.584	<0.001
PCT	ACE2	0.449	0.007
	IL-1RA	0.537	<0.001
	IL-10	0.438	0.009
	IL-18	0.553	<0.001
	IP-10	0.365	0.03
	MCP-1	0.389	0.02
	TNF-α	0.591	<0.001
PLT count	ACE2	-0.366	0.03
	IFN-γ	-0.351	0.038
	IL-1RA	-0.649	<0.0001
	IL-8	-0.569	<0.001
	IL-10	-0.566	<0.001
	IL-18	-0.357	0.035
	IP-10	-0.462	0.005
	MCP-1	-0.452	0.006
	TNF-α	-0.662	<0.0001
